# Community-driven secondary distribution of COVID-19 rapid antigen home tests in high-vulnerability Chicago neighborhoods: a community case study

**DOI:** 10.3389/fpubh.2026.1792442

**Published:** 2026-06-10

**Authors:** Elizabeth Lynch, Yolanda Cartwright, Lelia H. Chaisson, Latania K. Logan, Steve M. Epting, Elizabeth Davis, Elizabeth Avery, Tami Olinger, Angela Moss, Sumihiro Suzuki

**Affiliations:** 1 Department of Family and Preventive Medicine, Rush University Medical Center, Chicago, IL, United States; 2 Division of Infectious Diseases, College of Medicine, University of Illinois at Chicago, Chicago, IL, United States; 3 Department of Pediatrics, Rush University Medical Center, Chicago, IL, United States; 4 Hope Community Church, Chicago, IL, United States; 5 General Internal Medicine, Rush University Medical Center, Chicago, IL, United States; 6 Community, Systems and Mental Health Nursing, Rush University Medical Center, Chicago, IL, United States

**Keywords:** African American communities, community-based participatory research, COVID-19 testing, health equity, rapid antigen test

## Abstract

African American communities on Chicago’s South and West Sides, many facing high levels of social vulnerability, experienced disproportionate COVID-19 burden driven in part by limited access to diagnostic testing early in the pandemic. In partnership with the Alive Faith Network (AFN), a longstanding coalition of African American churches and academic partners, we implemented a participant-driven model to expand access to rapid antigen home tests during October 2021–August 2022. Community members distributed 8,386 rapid antigen home tests through their social networks, reaching many individuals who were young, male, and unvaccinated. Survey data were received for 1,555 test kits (18%), representing 1,075 unique individuals, and 602 test results were received. Follow-up calls revealed high acceptability and willingness to test again. Despite a low response rate, this case study highlights how decentralized, trust-based distribution models can overcome structural and behavioral barriers to early detection and extend public health reach into populations historically under-served by conventional approaches, highlighting the value of trusted community infrastructures in advancing health equity.

## Introduction

The COVID-19 pandemic exposed longstanding structural inequities in health across the United States. In Chicago, African American communities on the South and West sides, neighborhoods which experience high levels of social vulnerability due to historic and ongoing segregation and disinvestment, suffered profoundly higher COVID-19 morbidity and mortality than White communities (1). These patterns are consistent with a large national evidence base demonstrating that neighborhood-level social vulnerability is one of the strongest and most consistent predictors of COVID-19 outcomes in the United States. Across counties and cities, higher social vulnerability has been repeatedly associated with increased COVID-19 incidence, greater hospitalization severity, and higher mortality, with each decile increase in neighborhood disadvantage corresponding to substantial increases in deaths and severe illness (2–5).

Retrospective analyses of the first COVID-19 epidemic wave in Illinois demonstrate that these disparities were driven primarily by structural and social conditions, including residential segregation and occupational exposure, rather than biological susceptibility (6–8). More than three-quarters of the racial mortality gap was attributable to higher infection incidence, not increased risk of death once infected (6). Together, these findings underscore that effective mitigation in these communities required strategies capable of identifying infections early and disrupting transmission within specific social and occupational networks, rather than relying solely on downstream clinical treatment.

The testing infrastructure needed to support early detection was largely absent from the communities most affected during the critical early phase of the pandemic. Geographic analyses show that as the proportion of Black residents in a neighborhood increased, availability of testing sites declined, producing “testing deserts” that closely mirrored historical patterns of segregation (9). Although state and local efforts expanded testing capacity later in 2020 (10), testing access lagged behind transmission in Black neighborhoods (6). The consequence of poor testing access is visible in the testing results: where testing was least available, test results were disproportionately likely to be positive, indicating that transmission was far outpacing detection.

When testing access is insufficient, cases with mild or no symptoms — who have the least compelling reason to seek out a scarce testing resource — are the least likely to be tested and identified and continue to transmit the virus. Persistently high test positivity is not a sign that the virus is being contained but rather reflects a testing gap: the infections being counted represent only a fraction of those circulating in the community. This dynamic contributed to sustained transmission in Chicago’s most vulnerable neighborhoods despite ongoing testing efforts. Spatial analyses of Chicago demonstrated that clusters of high test positivity were co-located in areas of high social vulnerability on the West and South Sides, with a one-standard deviation increase in neighborhood social vulnerability associated with approximately 40% higher test positivity (11). Parallel findings from New York City reinforce this pattern: the proportion of positive tests declined as the proportion of White residents increased, with the highest positivity observed in non-White neighborhoods (12).

Beyond the elevated exposure risk facing essential workers, low-income residents in these communities faced significant opportunity costs to seeking testing. Costs such as lost wages, transportation, and time often outweighed the perceived benefits of testing particularly in the absence of severe symptoms, further suppressing early detection and enabling continued spread (13).

This testing environment disproportionately failed to detect infections among younger residents. Early pandemic surveillance data from Chicago during 2020 indicated that testing frequency increased with age, leaving pediatric and young adult populations markedly under-tested, and positivity rates remained consistently higher among younger residents (14). Younger adults were less likely to qualify for early testing, which was based on symptom severity, and significantly more likely to avoid testing because a positive result threatened economic stability (6). Individuals under 35 were more likely to cite inability to miss work following a positive test as a deterrent to testing. Consequently, testing behavior in this population became largely reactive rather than preventive, failing to prevent asymptomatic and pre-symptomatic transmission among younger, mobile workers who contributed disproportionately to transmission within these high-incidence communities (13).

At the same time, vaccination coverage in Chicago reflected a parallel inequity. Unlike Hispanic communities, where targeted outreach efforts led vaccination rates to match or exceed those of White residents, African American residents experienced a stark and persistent vaccination gap throughout the rollout (15–17). By late 2021, only 55.2% of African American residents had completed a vaccine series compared with approximately 70% of White and Hispanic residents, leaving majority-African American neighborhoods with the lowest levels of population protection relative to their high COVID-19 mortality burden (15, 18, 19). Across the pandemic, African American residents remained the least likely of all racial and ethnic groups to be vaccinated (2, 18).

Beyond logistical barriers, medical mistrust, rooted in historical abuses and ongoing structural racism, further constrained engagement with clinical testing, as well as vaccination, among African American residents (14, 15, 20). Even when geographically accessible, testing sites requiring interaction with healthcare institutions frequently failed to overcome this trust barrier (15).

To address these intersecting challenges, we leveraged our 15-year community partnership with the Alive Faith Network (AFN), a coalition of African American pastors working with academic researchers to advance health equity in Chicago. In collaboration with the AFN, we implemented a 21-month in-person COVID testing program across churches and community sites in African American neighborhoods in Chicago identified as “testing deserts” early in the pandemic (32). The program delivered 4,005 polymerase chain reaction (PCR) tests to 1,847 individuals. Testing uptake at church sites was consistently lower than at community locations (7.6 vs. 12.5 tests per day), in part due to low in-person church attendance, and overall demand fluctuated with vaccine rollout and increasing availability of home tests. Although expansion from churches to higher-traffic community sites improved reach, the model remained dependent on centralized access points that imposed transportation and scheduling burdens.

With the authorization of rapid antigen tests for home use in 2021, our AFN partners saw the opportunity to empower residents to distribute tests directly within their own social networks. We anticipated that this secondary distribution model could reduce structural barriers to testing, such as transportation and time constraints, while also helping to overcome medical mistrust by allowing people to receive and use tests through trusted social relationships rather than formal healthcare settings. While those initially engaged through community outreach might resemble the older, more civically involved populations typical of public health programs, their social ties could include younger, less vaccinated, and historically under-reached individuals who may have been more likely to be driving transmission.

This study evaluates the implementation of a peer-led secondary distribution program using the Ellume® home COVID-19 test (21) in African American communities in Chicago. We assess the demographic distribution of the intervention to determine whether secondary distribution expanded public health reach beyond traditionally engaged populations and into higher-risk social networks. By comparing individuals who initially received tests through community outreach with those who later obtained them via social networks, we evaluated whether a social-network-based approach could overcome the structural and behavioral barriers that limit conventional testing and extend reach to populations at elevated risk who are often missed by traditional strategies.

## Methods

### Context

The intervention was implemented between October 2021 and August 2022 in predominantly African American neighborhoods on Chicago’s West and South Sides, areas that have experienced historic disinvestment. These areas were among those with the city’s highest early pandemic mortality rates and lowest testing access (22), potentially leading to delays in diagnosis, with individuals often seeking care when symptoms were more advanced, further increasing morbidity and mortality.

Twenty distribution sites were used, including 10 churches and 10 community organizations/events, including food pantries, community-based organizations, and community celebrations. Sites were chosen from those affiliated with our in-person testing program conducted from December 2020 to August 2022 (32) and were selected based on high engagement and foot traffic during program implementation. Most sites (18/20) were located on Chicago’s West side reflecting both the geographic concentration of AFN partner churches and community organizations in that area and the neighborhoods with the highest early pandemic COVID-19 mortality rates and lowest testing access citywide.

## Key programmatic elements

### Community-academic collaborative development

During our in-person testing program, AFN churches served as trusted hubs for information, testing, and resource distribution, creating an established infrastructure that supported both feasibility and acceptability of a decentralized testing model. Building on this foundation, AFN partners proposed empowering residents to distribute rapid antigen tests within their own social networks to increase testing uptake, facilitate earlier detection, and reduce transmission in high-risk, underserved neighborhoods, particularly among individuals less likely to access traditional healthcare services. We collaborated with AFN leaders to co-develop the implementation strategy, communication materials, and distribution approach, ensuring that the model reflected community priorities and leveraged local relationships.

A community coordinator was established at each distribution site. Research staff trained community coordinators to implement the program and address questions from potential participants. The training covered an overview of the test kit contents, documentation and tracking procedures, testing guidance, and the registration and testing process using the Ellume® app.

### Secondary distribution model

For this study, we define “secondary distribution” as a community-driven strategy in which individuals initially engaged through outreach (“distributors”) were provided rapid antigen test kits to use themselves and share within their existing social networks (e.g., family, friends, neighbors, fellow congregants). This approach leverages existing social relationships to extend program reach beyond the individuals initially enrolled – a model with established precedent in HIV self-test distribution, where peer-based secondary distribution has been shown to reach individuals with low healthcare engagement who are unlikely to access facility-based testing (23–25). Recipients of the tests were also invited to enroll as distributors, allowing informal chains of outreach to grow organically within community networks.

### Testing protocol

We used the Ellume® COVID-19 Home Test, the first over-the-counter diagnostic test authorized by the FDA (in December 2020) that could be performed entirely at home without a prescription. The Ellume® test detects SARS-CoV-2 proteins with high sensitivity in both asymptomatic (91%) and symptomatic (96%) individuals (21). The Ellume® tests used in this study were not affected by the voluntary recall issued in October 2021 related to manufacturing issues that increased false-positive rates; all tests distributed in this program were from unaffected lots.

Participants self-collected a mid-turbinate nasal swab following step-by-step testing instructions provided through the Ellume® smartphone app. The swab was inserted into a Bluetooth-connected analyzer, and results were delivered in about 15 min.

Participants registered for the study electronically and completed a brief survey assessing demographics, vaccination status, symptoms, and testing history. Electronic consent was obtained from all registered participants (distributors and recipients) who chose to participate in research data collection prior to testing. Registration, consent, and survey data were collected via SnapSurveys (Version 11); test results were captured separately through the Ellume® app and matched to participants via unique ID. Each distributor received up to ten tests, illustrated instructions, and a $20 incentive to offset the time and effort involved in distributing tests within their social networks. Recipients were not incentivized but could receive the $20 incentive if they elected to enroll as a distributor. The test protocol included:

1. Registration: Via mobile device and unique QR code affixed to the test kit to confirm age (≥16 years required for self-testing) and generate a participant ID;
2. Consent and Survey: Electronic consent including information about how participant data would be used (including test results), followed by a brief pre-test survey using QR code technology;
3. Testing: Participants were connected to the Ellume® app via a link at the end of the survey or independently where they followed an in-app instructional video to perform the test, with results displayed and recorded electronically after 15 min.

Results were recorded automatically within the Ellume® app at the time of testing, including the test result and the date and time of use. Access to these data by the research team required that participants had registered and consented via SnapSurveys directly prior to conducting the test. For registered participants, test results and associated timestamps were provided to the study team via secure download from the product manufacturer (21). Although registration and survey participation were encouraged, they were not required to use the test, and individuals who did not register could use the Ellume® app without their data being shared with the research team. Once the tests were distributed to partner sites, the study team had no mechanism to follow up on data collection except through distributors who provided valid contact information. Missing data were not imputed; analyses were conducted on participants with complete data for each variable, which may further limit generalizability.

### Outreach and engagement strategy

Community coordinators at each distribution site approached attendees directly to explain the program and invite participation, supported by recruitment flyers advertising free at-home tests that highlighted ease of use and practical situations where testing was beneficial: before school or camp, visiting high-risk family members, traveling, or attending large gatherings. Individuals who expressed interest received a brief overview of the study, were informed that participation involved distributing up to 10 rapid antigen tests within their social networks and were directed to complete electronic registration via a QR code (connecting them to the SnapSurvey) before using the Ellume® app to take the test. Beyond the minimum age requirement (≥16 for self-testing), there were no additional eligibility criteria; all community members who agreed to distribute and use the tests were enrolled.

We also developed a social media campaign called “COVID is a Test” to increase awareness of at-home testing and support community-led distribution efforts. Delivered through platforms like Instagram and Facebook, the campaign featured culturally tailored content for African American audiences, including educational messages, testimonials and community stories, and messaging emphasizing resilience, collective responsibility, and the importance of protecting one’s household and neighborhood. A total of 9,334 people were reached via social media accounts, primarily Instagram. The audience reached was primarily young (60% between 18 and 34 years of age) and slightly more male (54%) than female (46%). Social media posts directed community members to our website which provided information on free at-home test pick-up locations and invited individuals to participate in the research study. Pick-up locations included AFN churches and other community-based organizations and events in the target neighborhoods. Website traffic was predominantly Illinois-based (76%), with the majority originating from zip codes within target areas on Chicago’s south and west sides (62%).

## Results

### Outcomes

All reported outcomes relate specifically to the implementation and evaluation of the secondary distribution model described in the “Key Programmatic Elements” section.

#### Program implementation metrics

**Figure 1:**
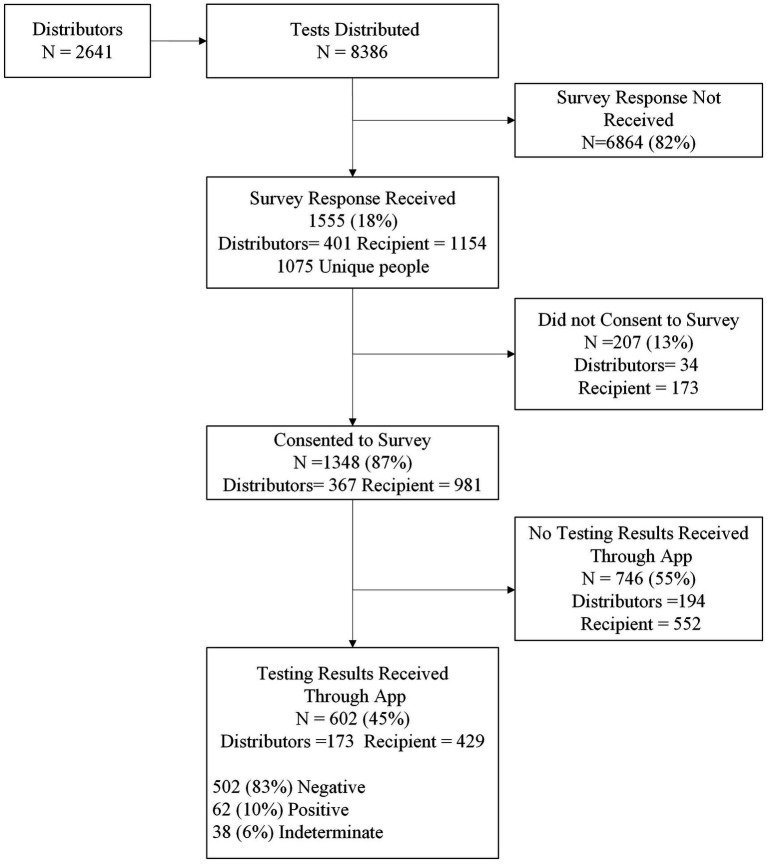
Intervention flow diagram.

We successfully recruited 2,641 individuals to collect tests for distribution (henceforth called “distributors”), 2,152 from churches, 377 from community-based organizations and events, and 112 from unspecified locations, who collectively distributed 8,386 at-home COVID-19 tests (3.2 tests distributed per person) (Figure 1). The median number of tests distributed per person was 2 (interquartile range: 2–4), indicating that half of distributors shared between 2 and 4 tests. A subset of distributors shared the maximum of 10 tests, while others retained tests for personal use. Of the 8,386 tests distributed, we received survey responses linked to 1,555 test kits (18%) from 1,075 unique individuals (some individuals completed the survey multiple times for different tests): 401 distributors and 1,154 individuals who received a test (henceforth called recipients). Among these, 1,348 completed the full survey. We received COVID-19 test results from 602 individuals who completed the survey (7% of tests distributed). Of those, 173 (29%) were from distributors and 429 (71%) were from recipients.

#### Participant characteristics

Differences between unique distributors and recipients were examined using a t-test for age and chi-square tests for all categorical variables. No adjustment for multiple comparisons was made given the exploratory, descriptive nature of these analyses. Missing data were handled by excluding individuals with missing values from the denominator for each variable, as reflected in the variable-specific denominators reported in Table 1.

**Table 1:** Characteristics of unique participants and other survey results.

Characteristics	Total unique participants	Test distributor	Test recipient	*p*-value
Unique participants	*N* = 1,075	*N* = 275	*N* = 800	
Age, Mean (SD)	39.7 (19.9)	48.2 (17.5)	36.8 (19.8)	<0.001^a^
Age group, *N* (%)				<0.001^b^
<18	160 (14.9)	4 (1.4)	156 (19.5)	
18–40	389 (36.2)	90 (32.7)	299 (37.4)	
40–64	399 (37.1)	130 (47.3)	269 (33.6)	
> 65	127 (11.8)	51 (18.6)	76 (9.5)	
Consented, *N* (%)	929 (86.4)	247 (89.8)	682 (85.2)	
Gender, *N* (%)^c,d^				0.012^b^
Male	311 (33.6)	67 (27.1)	244 (35.9)	
Female	615 (66.4)	180 (72.9)	435 (64.9)	
Hispanic, *N* (%)^c^	47 (5.1)	14 (5.7)	33 (4.8)	0.610^b^
Race, *N* (%)^c^				0.266^b^
African American	779 (83.9)	215 (87.0)	564 (82.7)	
White	84 (9.0)	17 (6.9)	67 (9.8)	
Other	66 (7.1)	15 (6.1)	51 (7.5)	
Vaccinated, *N* (%) ^c,d^	722 (77.9)	216 (87.8)	506 (74.3)	<0.001^b^
Tested positive in the past, *N* (%)^c^	314 (33.8)	94 (38.1)	220 (32.3)	0.099^b^
COVID symptoms, *N* (%)^c^	412 (44.3)	92 (37.2)	320 (46.9)	0.008^b^
Friend\Family tested positive, *N* (%)^c^	459 (49.4)	129 (52.2)	330 (48.4)	0.301^b^

SD, standard deviation. Missing data were not imputed; analyses were conducted on participants with complete data for each variable. ^a^*T*-test. ^b^Chi-Square Test. ^c^Percentage of consented. ^d^Percentage of consented with missing data removed from denominator. Gender denominator = 926 (3 missing from test recipient), Vaccinated denominator = 927 (1 missing from test recipient; 1 missing from distributor).

Of the 1,075 unique individuals who submitted a survey, participants were predominantly African American (84%), with a mean (SD) age of 39.7 (19.9) years (Table 1). Compared to distributors, recipients were younger (mean age 37 vs. 48 years, *p* < 0.001), more likely to be male (36% vs. 27%, *p* = 0.012), less likely to be vaccinated (74% vs. 88%, *p* < 0.001), and more likely to be experiencing COVID-19 symptoms (47% vs. 37%, *p* = 0.008). Notably, 19% of recipients were under 18 years of age.

Despite a low survey response rate, there is some evidence that this intervention successfully reached populations identified as facing the greatest barriers to early detection and protection, particularly younger individuals and those who were unvaccinated. Those groups have been shown to be less likely to engage in COVID-related care and preventive services (26). More complex modeling was not conducted due to the low response rate, which could have introduced bias. Additionally, very little information was available about test recipients beyond those who completed the survey, so missing data imputation was not performed.

#### Testing outcomes

Among participants who completed the survey and had results returned via the Ellume® app (*n* = 602), a majority of results were negative (*n* = 502, 83%), 62 (10%) were positive, and 38 (6%) were indeterminate (Figure 1). Our positivity rate of 10% was higher than the citywide test positivity rates during the study period, which ranged from 2.1 to 7.7% (1), with fluctuations due to local surges. While these estimates are not directly comparable since citywide rates were largely based on PCR testing conducted in clinical or public testing settings and our program used rapid antigen tests in a community-based, self-testing context, the higher positivity observed in our program may still suggest that testing reached individuals at elevated risk of infection.

#### Follow-up engagement

We conducted follow-up phone calls with test distributors to assess uptake and experience. We had valid phone numbers for 1,795 distributors and successfully reached 597 (33%). Among those reached, over half (*n* = 334, 56%) reported giving tests away to others and using a test themselves (*n* = 328, 55%). Overall, this corresponds to approximately 19% of all distributors with valid phone numbers confirming test distribution. Among those who used a test, most said the instructions were clear (*n* = 312, 95%) and that they would take another test in the future (*n* = 302, 92%). Although follow-up was limited by a modest response rate, these findings indicate the feasibility and acceptability of community-driven test distribution in this population.

## Discussion

This community-driven testing initiative demonstrates the potential of trusted local networks to extend public health resources to areas where need is greatest. In neighborhoods where social vulnerability has been repeatedly shown to predict higher COVID-19 incidence, hospitalization severity, and mortality, prevailing clinic- and site-based testing strategies were poorly aligned with the structural and behavioral realities shaping early detection and transmission control, as reflected in persistently high test positivity and delayed case identification in these communities. By shifting diagnostic access from centralized institutions to trusted community relationships, our intervention directly addressed the structural, behavioral, and trust-related barriers faced by socially vulnerable communities.

Notably, this intervention occurred during a period marked by social distancing, intermittent public health restrictions, pandemic fatigue, and social isolation, all of which disrupted healthcare-seeking behaviors and routine engagement with traditional healthcare systems, particularly among populations facing structural barriers to care. Therefore, decentralized testing approaches embedded within trusted community networks may have been especially valuable by enabling access to testing without requiring formal healthcare interactions, transportation, or visits to centralized testing sites.

A growing body of evidence suggests that community-based outreach grounded in trust and cultural relevance is more effective than approaches led by health systems for reaching underserved populations (26, 27). Community-partnered testing models have proven especially critical in high-risk African American communities, where medical mistrust and structural inequities limit healthcare engagement (27, 28). Reviews of large-scale COVID-19 testing efforts have consistently found that partnerships with trusted community organizations lead to greater uptake than outreach conducted solely through health systems (28, 29). Our findings reinforce and extend this evidence base. In the context of COVID-19, such approaches are particularly critical in African American communities, where structural disinvestment, occupational exposure, and medical mistrust work together to inhibit early engagement with preventive services. Prior community-based COVID-19 testing initiatives, including our own earlier work conducted in partnership with faith organizations (32), used churches as trusted venues for centralized testing or outreach (27, 28). The current model extended this approach by enabling secondary distribution of rapid tests through participants’ social networks, allowing testing access to extend beyond the original community setting.

The elevated test positivity observed among program participants exceeded citywide rates. Although differences in test type, testing indications, and underlying populations limit direct comparison, these findings suggest that this decentralized model has the potential to reach individuals at higher risk of infection who were not being captured by conventional testing systems.

The demographic patterns observed in this study highlight the value of a social network-based approach. Although individuals initially engaged through community outreach tended to be older and more likely to be vaccinated, characteristics typical of participants in public health programs, the tests were subsequently distributed through social ties to individuals who were younger, more likely to be male, and less likely to be vaccinated. These groups have been shown to engage less frequently in COVID-related care and preventive services (26). Despite a modest survey response rate, the intervention reached populations facing documented barriers to early detection and protection.

Comparison with Bien-Gund et al. (26), a randomized clinical trial of secondary COVID-19 self-test distribution through four federally qualified health centers (FQHCs) in Philadelphia, further contextualizes our findings and underscores the central role of trust infrastructure in decentralized testing models. In that trial, 776 adults were randomized to receive either five self-test kits or five clinic referral cards to distribute within their social networks. Despite this structured clinical support, the primary outcome, at least two network contacts with confirmed testing at 8 weeks, was achieved by only 1.3% of participants in the self-test group versus 0.5% in the control group, a non-significant difference (*p* = 0.45). By end of study, only 5.2% of self-test recipients had a single confirmed network contact tested. Bien-Gund et al. noted that self-reported distribution was substantially higher than objectively confirmed testing, with 86.9% of participants reporting giving out at least one kit, a discrepancy they attribute to measurement challenges inherent to decentralized designs rather than participant unwillingness.

Our program differed in three important structural respects. First, it was embedded within a 15-year community-faith partnership rather than a clinical encounter, engaging participants through relationships that pre-existed the pandemic and that carried institutional trust. Second, financial incentives were provided to distributors, and extended to recipients who became distributors, a feature absent from the Bien-Gund trial. Third, our program achieved substantially greater scale: more than 2,600 participants and over 8,000 tests distributed across 20 community sites. Critically, our demographic findings suggest that this community-embedded model reached individuals who were younger, less vaccinated, and less connected to formal health systems, populations that FQHC-based recruitment is structurally less likely to engage. Taken together, these comparisons suggest that secondary distribution as a mechanism is promising, but that its effectiveness may depend on the trust infrastructure through which it is deployed rather than the distribution approach alone.

Secondary distribution of self-tests as a public health strategy was pioneered in HIV testing, where a substantial body of work established that peer-led redistribution through social networks can reach individuals with low healthcare engagement, including those who are younger, less connected to formal health systems, and at elevated risk of transmission (23, 24). Community-based peer distribution models have been particularly notable for this reach: in a pilot trial among Ugandan fishing communities, Choko et al. (30) found that recipients of peer-distributed HIV self-tests were younger than those who distributed to them, and nearly one in four had not previously been tested, a pattern closely echoing our finding that secondary COVID-19 test recipients were younger and less vaccinated than the distributors who reached them. Across this literature, self-reported distribution has consistently exceeded objectively verified test use, reflecting the inherent measurement challenges of decentralized models rather than program failure (31). The present study extends this peer distribution approach to COVID-19 in a community-faith context, and its findings echo both the promise and the measurement challenges documented in the HIV literature.

Notably, although tests were distributed through both churches and community-based organizations, approximately 80% were delivered through church partners. This likely reflects the depth and longevity of our relationships with faith institutions, which predated the pandemic by more than a decade. These findings reinforce the importance of long-standing community partnerships in enabling rapid, large-scale public health responses, particularly in communities where mistrust in external institutions remains high.

Most importantly, this study demonstrates that decentralized, relationship-driven testing strategies can offer a complementary approach to conventional models in underserved communities. Integrating testing into existing social networks aligns public health efforts with actual transmission dynamics, enabling earlier detection and potentially reducing morbidity and mortality.

## Implications for public health practice

The implications of this work extend beyond COVID-19. The same trust-based, network-oriented framework can be applied to other public health priorities, including vaccination, chronic disease screening, and distribution of preventive resources in communities facing persistent structural barriers. Strengthening and sustaining community partnerships like the AFN is therefore not only an imperative to promote equity but a core component of resilient public health infrastructure.

Social network-based public health strategies may be particularly important during pandemics and other emergencies when healthcare systems are strained, public trust may be weakened, and rapid dissemination of preventive resources is needed. Unlike centralized models, secondary distribution approaches leverage trusted community relationships to extend reach beyond individuals already connected to healthcare systems. These approaches may be especially effective for populations facing structural barriers to care and may have broader relevance for future emergency preparedness and public health crises.

## Limitations

This study has several limitations. Survey participation was voluntary, and the survey and test result systems operated on separate platforms: completing one did not populate the other, and recipients could use the Ellume® test without engaging with the survey. As a result, the 18% survey response rate reflects the proportion of test recipients who voluntarily chose to participate in research, not dropout from an enrolled cohort. While this low response rate raises the possibility of selection bias, the most likely direction of that bias works against our findings rather than in support of them. Individuals who are younger, less vaccinated, and less connected to the healthcare system are less likely to navigate a two-platform digital data collection process with no financial incentive. Survey respondents among secondary recipients therefore probably over-represent the most engaged members of that group, meaning our finding that recipients were younger, less vaccinated, and less healthcare-engaged than distributors is likely a conservative estimate of the true disparity. A separate selection concern is that distributors may have preferentially shared tests with their most health-engaged social contacts; this cannot be assessed from available data and represents a constraint on characterizing downstream reach. In addition, use of a smartphone-based testing platform introduced technology-related barriers that may have excluded some residents and follow-up among distributors was constrained by high residential mobility and inconsistent contact information.

The program also has inherent design limitations. As a community service initiative, the program lacked a formal control group and could not fully map downstream distribution pathways. Because the secondary distribution approach was implemented as community outreach rather than a probability-based sampling design, results are descriptive and implementation-focused and should not be interpreted as representative of the broader community. We were also unable to compare our COVID-19 incidence rate with community incidence rates stratified by test type (e.g., PCR, point-of-care clinic testing, at-home testing) limiting interpretability of positivity data in the broader epidemiological context. Finally, comparisons between distributor and recipient subgroups were exploratory and descriptive, and no correction for multiple comparisons was applied.

## Lessons learned and recommendations for replication

Implementation revealed several practical lessons that may guide future programs. First, integrating the study survey directly within the test app from the outset would improve data capture. In this program, the survey and the Ellume® app operated as separate platforms, requiring participants to navigate between two digital tools, a barrier that likely contributed to the low response rate. Programs using app-connected diagnostics should negotiate data-sharing agreements with manufacturers in advance to enable seamless, consent-based data capture within a single interface.

Second, collecting more detailed information from distributors at enrollment, including their intended distribution network, relationship types, and estimated network size, would improve both program monitoring and interpretation of downstream reach. Retrospectively, we were unable to characterize the social networks through which tests traveled, limiting our ability to assess how far secondary distribution extended beyond initial recipients.

Third, community coordinators were central to program implementation, and adequate training and logistical support is essential. Future programs should budget sufficient time for pilot testing of the full participant-facing workflow with a diverse range of community members before broad rollout.

For communities seeking to replicate this model, the core requirements are: an established, trust-based community partnership capable of mobilizing participants at scale; a sufficient supply of test kits with clear, literacy-appropriate instructions; a simple registration or data-capture mechanism accessible via smartphone; a modest participant incentive (in this case, $20 per distributor); and dedicated program coordination staff. The core distribution model does not inherently require research infrastructure and could be adapted for public health practice settings with appropriate coordination resources. Realizing this potential at scale will require sustained investment in community-academic partnerships and flexible data systems, and the trust that enables these programs is built over years, not months, underscoring the importance of maintaining such partnerships as a standing public health capacity.

## Conclusion

In communities where social vulnerability and structural inequities shape chronic disease risk, conventional public health approaches alone may be insufficient. This study suggests that when diagnostic tools are embedded within trusted social networks, public health interventions can reach populations historically excluded from early detection and prevention. Community-driven, decentralized strategies such as this one may represent a promising and adaptable framework for advancing health equity, with relevance for both post-pandemic public health practice and future public health emergencies.

## Data Availability

The raw data supporting the conclusions of this article will be made available by the authors, without undue reservation.

## References

[1] Chicago Department of Public Health. Covid-19 dashboard. (2021). Available online at: https://www.chicago.gov/content/city/en/sites/covid-19/home/covid-dashboard.html

[2] Karaye IM Horney JA. The impact of social vulnerability on COVID-19 in the U.S.: an analysis of spatially varying relationships. Am J Prev Med. (2020) 59:317–25. doi: 10.1016/j.amepre.2020.06.006, 32703701 PMC7318979

[3] Tipirneni R Schmidt H Lantz PM Karmakar M. Associations of 4 geographic social vulnerability indices with US COVID-19 incidence and mortality. Am J Public Health. (2022) 112:1584–8. doi: 10.2105/AJPH.2022.307018, 36108250 PMC9558191

[4] Tipirneni R Karmakar M O'Malley M Prescott HC Chopra V. Contribution of individual- and neighborhood-level social, demographic, and health factors to COVID-19 hospitalization outcomes. Ann Intern Med. (2022) 175:505–12. doi: 10.7326/M21-2615, 35188790 PMC8982172

[5] Patterson EJ Johnson LT. Structural inequality and COVID-19 mortality in Chicago: an ecological analysis. J Racial Ethn Health Disparities. (2023) 10:2620–9. doi: 10.1007/s40615-022-01440-1, 36348182 PMC9643901

[6] Holden TM Simon MA Arnold DT Halloway V Gerardin J. Structural racism and COVID-19 response: higher risk of exposure drives disparate COVID-19 deaths among black and Hispanic/Latinx residents of Illinois, USA. BMC Public Health. (2022) 22:312. doi: 10.1186/s12889-022-12698-9, 35168585 PMC8845334

[7] Anderson KF Lopez A Simburger D. Racial/ethnic residential segregation and the first wave of SARS-CoV-2 infection rates: a spatial analysis of four U.S. Cities Sociol Perspect. (2021) 64:804–30. doi: 10.1177/07311214211041967, 38603057 PMC8404417

[8] Kim SJ Bostwick W. Social vulnerability and racial inequality in COVID-19 deaths in Chicago. Health Educ Behav. (2020) 47:509–13. doi: 10.1177/1090198120929677, 32436405 PMC8183499

[9] Asabor EN Warren JL Cohen T. Racial/ethnic segregation and access to COVID-19 testing: spatial distribution of COVID-19 testing sites in the four largest highly segregated cities in the United States. Am J Public Health. (2022) 112:518–26. doi: 10.2105/AJPH.2021.306558, 35196059 PMC8887160

[10] English K Lei U Shipman-Amuwo F Burkey M Msp Gonzalez JG . Community-based testing for SARS-CoV-2 - Chicago, Illinois, may-November 2020. MMWR Morb Mortal Wkly Rep. (2021) 70:707–11. doi: 10.15585/mmwr.mm7019a4 33983914 PMC8118149

[11] Bilal U Tabb LP Barber S Diez Roux AV. Spatial inequities in COVID-19 testing, positivity, confirmed cases, and mortality in 3 U.S. cities: an ecological study. Ann Intern Med. (2021) 174:936–44. doi: 10.7326/M20-3936, 33780289 PMC8029592

[12] Lieberman-Cribbin W Tuminello S Flores RM Taioli E. Disparities in COVID-19 testing and positivity in New York City. Am J Prev Med. (2020) 59:326–32. doi: 10.1016/j.amepre.2020.06.005, 32703702 PMC7316038

[13] Perry BL Aronson B Railey AF Ludema C. If you build it, will they come? Social, economic, and psychological determinants of COVID-19 testing decisions. PLoS One. (2021) 16:e0252658. doi: 10.1371/journal.pone.0252658, 34260602 PMC8279331

[14] Kim SJ Watson K Khare N Shastri S Da Goia Pinto CL Nazir NT. Addressing racial/ethnic equity in access to COVID-19 testing through drive-thru and walk-in testing sites in Chicago. Med Res Arch. (2021) 9:2430. doi: 10.18103/mra.v9i5.2430, 34109272 PMC8186439

[15] Kim SJ McWhirter N Duong K Khare MM Giles WH Basu S . COVID-19 vaccine policy implementation and differential vaccine uptake trajectories in Chicago communities. J Public Health Manage Pract. (2024) 30:E21–e30. doi: 10.1097/PHH.0000000000001841, 37966958 PMC10723817

[16] DiVirgilio L Boshara A Hunt BR Jacobs J Just K Johnson AK. A community-informed approach to COVID-19 vaccine roll-out in under-served areas in Chicago. Front Public Health. (2022) 10:863125. doi: 10.3389/fpubh.2022.863125, 35795703 PMC9251185

[17] Keegan G Zhu M Paz M Kang H Patel A Baig AA. Neighborhood-level factors associated with COVID-19 vaccination rates: a case study in Chicago. BMC Public Health. (2024) 24:889. doi: 10.1186/s12889-024-18352-w, 38528490 PMC10962191

[18] Zeng S Pelzer KM Gibbons RD Peek ME Parker WF. Association of zip Code Vaccination Rate with COVID-19 mortality in Chicago, Illinois. JAMA Netw Open. (2022) 5:e2214753. doi: 10.1001/jamanetworkopen.2022.14753, 35622360 PMC9142872

[19] Phillips B Baker L Faherty LJ Ringel JS Kranz AM. Mapping changes in inequities in COVID-19 vaccinations relative to deaths in Chicago, Illinois. Prev Chronic Dis. (2023) 20:E32. doi: 10.5888/pcd20.220319, 37115106 PMC10159334

[20] Tung EL Peek ME Rivas MA Yang JP Volerman A. Association of neighborhood disadvantage with racial disparities in COVID-19 positivity in Chicago. Health Aff. (2021) 40:1784–91. doi: 10.1377/hlthaff.2021.00695, 34724418 PMC8975623

[21] Ellume® COVID-19 Home Test - Instructions for Use. U.S. Department of Health and Human Services. Avaialble online at: https://www.fda.gov/media/144592/download

[22] Smith CS. “Multimodal Access to COVID-19 Testing Facilities in Chicago and Comparisons by Race/Ethnic Community and COVID-19 Vulnerability.” Presented at the City of Chicago Racial Equity Rapid Response Team (RERRT) Meeting, On-Line (2020).

[23] Masters SH Agot K Obonyo B Napierala Mavedzenge S Maman S Thirumurthy H. Promoting partner testing and couples testing through secondary distribution of HIV self-tests: a randomized clinical trial. PLoS Med. (2016) 13:e1002166. doi: 10.1371/journal.pmed.1002166, 27824882 PMC5100966

[24] Thirumurthy H Masters SH Mavedzenge SN Maman S Omanga E Agot K. Promoting male partner HIV testing and safer sexual decision making through secondary distribution of self-tests by HIV-negative female sex workers and women receiving antenatal and post-partum care in Kenya: a cohort study. Lancet HIV. (2016) 3:e266–74. doi: 10.1016/S2352-3018(16)00041-2, 27240789 PMC5488644

[25] Sithole N Koole O Sausi K Krows M Schaafsma T Van Heerden A . Secondary distribution of HIV self-testing kits to social and sexual networks of PLWH in KwaZulu-Natal, South Africa. A brief report. Front Public Health. (2022) 10:855625. doi: 10.3389/fpubh.2022.855625, 35570932 PMC9092373

[26] Bien-Gund CH Stephens-Shields AJ Acri T Dugosh K Gross R. Provision of COVID-19 self-test kits to patients for distribution to social contacts: a randomized clinical trial. JAMA Netw Open. (2025) 8:e2513708. doi: 10.1001/jamanetworkopen.2025.13708, 40465296 PMC12138724

[27] Maras SA Osinuga A Gallo IV Rodriguez A Corriveau E Milligan K . Qualitative evaluation of RADx-UP projects addressing COVID-19 testing disparities among underserved populations. Am J Public Health. (2024) 114:S410–5. doi: 10.2105/AJPH.2024.307632, 38547469 PMC11111372

[28] Hamilton-Burgess C Berkley-Patton J Allsworth J Bowe Thompson C Thompson FE Burgin T . The importance of community-based and community-partnered COVID-19 testing for reducing disparities among African American populations. Health Equity. (2024) 8:147–56. doi: 10.1089/heq.2022.0185, 38505761 PMC10949942

[29] Lane-Barlow C Thomas I. Experiences of health departments on community engagement and implementation of a COVID-19 self-testing program. J Public Health Manag Pract. (2023) 29:539–46. doi: 10.1097/PHH.0000000000001688, 36729971 PMC10198798

[30] Choko AT Nanfuka M Birungi J Taasi G Kisembo P Helleringer S. A pilot trial of the peer-based distribution of HIV self-test kits among fishermen in Bulisa, Uganda. PLoS One. (2018) 13:e0208191. doi: 10.1371/journal.pone.0208191, 30496260 PMC6264512

[31] Hensen B Schaap AJ Mulubwa C Floyd S Shanaube K Phiri MM . Who accepts and who uses community-based secondary distribution HIV self-testing (HIVST) kits? Findings from the intervention arm of a cluster-randomized trial of HIVST distribution nested in four HPTN 071 (PopART) communities in Zambia. J Acquir Immune Defic Syndr. (2020) 84:355–64. doi: 10.1097/QAI.0000000000002344, 32195749 PMC7340225

[32] Cartwright Y Logan L Chaisson L Suzuki S Epting S Davis E . Implementing a COVID-19 Testing Intervention in African American Testing Deserts in Chicago: An Academic–Community Partnership to Promote COVID-19 Health Equity. Manuscript under review at Journal of Racial and Ethnic Health Disparities. 10.1007/s40615-026-03067-y 42340644

